# Decoding the synergy: unveiling gradient boosting regression model for multivariate quantitation of pioglitazone, alogliptin and glimepiride in pure and tablet dosage forms

**DOI:** 10.1186/s13065-024-01351-8

**Published:** 2024-11-29

**Authors:** Mahmoud M. Elkhoudary, Aya A. Marie, Sherin F. Hammad, Mohamed M. Salim, Amira H. Kamal

**Affiliations:** 1 Department of Pharmaceutical Chemistry, Faculty of Pharmacy, Horus University-Egypt, New Damietta, 34517 Egypt; 2 Department of Pharmaceutical Analytical Chemistry, Faculty of Pharmacy, Tanta University, Tanta, 31527 Egypt; 3 Department of Pharmaceutical Analytical Chemistry, Faculty of Pharmacy, Mansoura University, Mansoura, 35516 Egypt; 4 Faculty of Pharmacy, Medical Campus of Tanta University, Elgeish Street, Tanta, 31111 Egypt

**Keywords:** Partial least squares, UV/Vis spectroscopy, Artificial neural networks, Extreme gradient boosting algorithm, Support vector regression

## Abstract

This study represents a comparison among the performances of four multivariate procedures: partial least square (PLS) and artificial neural networks (ANN) in addition to support vector regression (SVR) and extreme gradient boosting (XG Boost) algorithm for the determination of the anti-diabetic mixture of pioglitazone (PIO), alogliptin (ALG) and glimepiride (GLM) in pharmaceutical formulations with aid of UV spectrometry. Key wavelengths were selected using knowledge-based variable selection and various preprocessing methods (e.g., mean centering, orthogonal scatter correction, and principal component analysis) to minimize noise and improve model precision. XG Boost effectively enhanced computing speed and accuracy by focusing on specific spectral features rather than the entire spectrum, demonstrating its advantages in resolving complex, overlapping spectral data. The independent test results of different models demonstrated that XG Boost outperformed other methods. XG Boost achieved the lowest root mean squared error of prediction (RMSEP) and standard deviation (SD) values across all compounds, indicating minimal prediction error and variability. For PIO, XG Boost recorded an RMSEP of 0.100 and SD of 0.369, significantly better than PLS and ANN. For ALG, XG Boost showed near-perfect performance with an RMSEP of 0.001 and SD of 0.005, outperforming SVR and PLS, which had higher error rates. In the case of GLM, XG Boost also excelled with an RMSEP of 0.001 and SD of 0.018, demonstrating superior precision compared to the much higher errors seen in PLS and ANN. These results highlight XG Boost’s exceptional ability to handle complex, overlapping spectral data, making it the most reliable and accurate model in this study.

**Supplementary Information:**

The online version contains supplementary material available at 10.1186/s13065-024-01351-8.

## Introduction

Diabetes represents a serious wide spreading health problem in Egypt with an extensive effect on morbidity and death [1]. Egypt is the 9th top nation for the number of diabetes patients all over the world [1].

Alogliptin benzoate (ALG) is DPP-4 (dipeptidyl peptidase-4) inhibitor [2] thus leads to control the glycaemia [3].

Pioglitazone (PIO) is one of thiazolidinediones [4]. It is an agonist of PPAR (Peroxisome Proliferator-Activated Receptor) gamma. It is used to control type II diabetes and to decrease insulin resistance [4].

Glimepride (GLM) is one of the long-acting anti-diabetic agents used orally to decrease the level of blood sugar in type II diabetes [5].

Several analytical approaches were described for estimation of ALG [6–17], PIO [18–22] and GLM [23–27] alone and in combinations. Spectroscopic methods [6–11, 22, 25, 27] are simple, easy, rapid but depend on univariate analysis using single regression equation while the proposed multivariate methods eliminate the errors of single regression equations and provide more reliable results with high accuracy and robustness. Chromatographic methods [12–20, 23, 24, 26] are accurate, precise, and selective but need sophisticated instrumention as in HPLC in addition to time and reagent consumption as well as high cost. The proposed Multivariate methods save time and reagents, more ecofriendly than the chromatographic methods, and do not require any separation step prior its application.

This work pointed to challenge the ordinarily used multivariate chemometric techniques in treating the UV absorption data of exceptional mixtures that cannot be identified or measured with univariate spectrophotometric methods or common multivariate ones. The proposed models were PLS (partial least squares) and ANN (artificial neural networks) in addition to SVR (support vector regression) and XG Boost extreme gradient boosting algorithm. While the proposed mixtures for this challenge were (PIO: ALG) in a ratio of (30: 25) and (PIO: GLM) in a ratio of (30: 4). These mixtures are commercial dosage forms; Piompride^®^ (4/30) (4 mg GLM & 30 mg PIO) and Prandglim plus^®^ (25/30) (25 mg ALG & 30 mg PIO).

The utilization of chemometric models is usually accompanied by compromise between using the full spectrum features (wavelengths) and the computational power, preprocessing time, and model strength. Many preprocessing and data reduction paradigms are usually adopted to reach ideal wavelengths, bands or scores that best describe the spectral data with respect to analyzed components. Then the variable combination step comes that has great effect on prediction [23]. Even if less important variables are combined in the subset, they can achieve improved prediction [24, 25] so the concept of combined variable is announced into spectrum analysis. Whereas chromatographic methods are usually known for their superiority in separation and quantitation of components in their complex mixtures, these methods demand sophisticated equipment and expensive reagents used in large amounts are harmful to the environment. In opposition, the chemometric spectrophotometric techniques are cost and time saving compared to most chromatographic methods as well as do not need sophisticated instruments or any former separation step. Multivariate methods are more robust than univariate methods due to application of multiple regression at multiple wavelengths that gives more reliable results.

It is well known among chemometricians that it is necessary to build a chemometric model after measuring spectra related to training, validation, test and dosage form sets in one working period at the same day to avoid large amount of variability and noise as well as to increase method precision. However, this approach neglects the fact that method robustness and ability of method transfer are then limited and nearly unapplicable.

Non-linear spectral data present significant challenges, such as overlapping peaks, scattering, and spectral noise, and variations in sample preparation that complicate accurate quantification and discrimination of compounds as well as modeling of relationships between spectral features and analyte concentrations. Traditional linear models like PLS often fail under these conditions, leading to inaccurate predictions. Non-linear models, such as Support Vector Regression (SVR), are crucial as they effectively capture complex relationships in the data and reduce the impact of spectral noise, providing more accurate and robust predictions. Selecting appropriate multivariate models is essential to manage these complexities and achieve reliable results in complex spectral environments [28, 29].

XG Boost algorithms can be introduced as a candidate that offers several advantages in treating the non-linear data and extracting features from variables having noise and less information even with drugs with very low concentration or with very high spectral contribution. Numerous studies demonstrated that it improved the accuracy of prediction and accomplished noteworthy results for spectral analysis in various domains [32–34] but there is no work applies this approach to quantitative spectral analysis of pharmaceutical preparations as well as to conduct the comparison with ANN, SVR and PLS models. This work introduces XG-boost into determination of pharmaceutical preparations to measure concentration of components that have high degree of complexity in their UV spectra.

## Experimental

### Apparatus and software

The UV absorption spectra were recorded using Schimadzu (UV-1800, Kyoto, Japan) UV-VIS double beam spectrophotometer. Quartz cell (1 cm) was used. UV-Prop 2.33 software was utilized. Data analysis was performed by Matlab^®^ 9.8.0.123 (R2020a), Eigenvector PLS Toolbox 9.1 and MS Excel 356.

### Active pharmaceutical ingredients

**Fig. 1:**
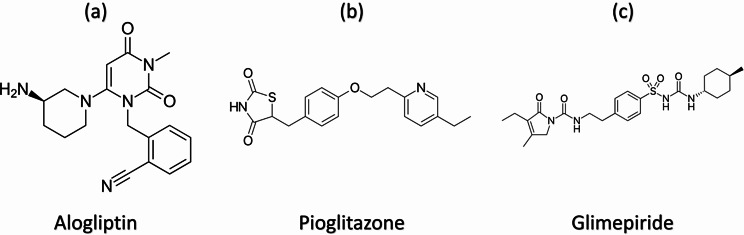
Chemical structures of Alogliptin (**a**), pioglitazone (**b**) and glimepiride (**c**)

Pure standard ALG (Fig. 1a) (99.30% purity), PIO (Fig. 1.b) (99.50% purity) and GLM (Fig. 1.c) (99.70% purity) were gifted from Sigma for Pharmaceutical Industries (Mubarak Industrial Zone, Quesna, Egypt).

Dosage forms from Egyptian markets are Piompride^®^ (4/30) contain (4 mg GLM and 30 mg PIO) with batch number (191294) from AVERROES PHARMA (6th Industrial Zone, Sadat City- Menofia, Egypt) and Prandglim plus^®^ (25/30) contain (25 mg ALG and 30 mg PIO) with batch number (2004983) from EVA PHARMA (Abdeen, Cairo, Egypt).

### Reagents

Methanol (analytical grade, Fisher, UK) was used.

### Preparation of stock and working standard solutions

Stock solutions of ALG and PIO were prepared by transferring 50 mg ALG and PIO into 50 mL separated volumetric flasks to obtain 1000 µg/mL of each. GLM stock solution was prepared by transferring 20 mg into 50 mL volumetric flask to obtain 400 µg/mL GLM. The working solutions 500 µg/mL ALG and PIO and 100 µg/mL GLM were prepared by suitable dilutions using methanol into 100 mL separated volumetric flasks.

### Piompride® and Prandglim plus® tablets

Ten tablets of either Piompride^®^ (4/30) or Prandglim plus^®^ (25/30) tablets were separately weighed, grinded and finally powdered with homogeneous mixing. A weight of ground tablets equivalent to (4 mg GLM & 30 mg PIO) and (25 mg ALG & 30 mg PIO), were respectively taken into two separate 100- mL volumetric flask. The powdered tablets dissolved using 75 mL methanol. Then the obtained solutions were sonicated for 20 min. After cooling, methanol was added up to the mark with. The final solutions were filtered, and the first portion of filtrates were rejected. Various aliquots were taken from each filtrate to obtain various concentrations of ALG, PIO and GLM by serial dilutions of the filtrates using the same solvent. UV spectra of these solutions were recorded using the procedures mentioned previously.

### Spectral data variable selection and preprocessing

Knowledge based variable selection was adopted to select the sensitive feature bands in spectrum or the wavelengths that were affected by the concentration of each drug. The variable selection approach employed was knowledge-based variable selection, which focused on identifying the most relevant and sensitive spectral regions that were directly influenced by the concentration of each drug (PIO, ALG, and GLM). This method relied on prior knowledge of the chemical and spectral characteristics of the compounds to guide the selection process, emphasizing wavelength regions where the analytes showed the most significant spectral responses. By targeting these specific feature bands, we aimed to maximize the model’s sensitivity and accuracy while minimizing noise and irrelevant information. Several wavelength combinations were systematically tested to determine the optimal set that provided the best predictive performance for each compound, enhancing the robustness and precision of the multivariate models used in this study. This approach ensured that the selected wavelengths were not only statistically significant but also chemically relevant, providing a clear advantage in accurately quantifying each drug within complex mixtures. Many wavelength combinations were tested for the three candidate components.

Spectral data were evaluated first by preprocessing methods to remove sources of variations such as base line drift, instrumental and environmental effects. Several preprocessing methods were tested including mean centering (MC), auto scale (AS) as well as orthogonal scatter correction (OSC) in addition to multiplicative scatter correction (MSC) and standard normal variants (SNV) to select methods with best results and minimum error.

Orthogonal scatter correction is based on PLS which gets rid of X-data variation which is not related to Y as OSC utilizes Y to create a filter of X so it provides useful information about the correction of X based on scores and loadings diagnostics [30].

OSC has better predictive ability in multicomponent analysis when compared to other models used non-filtered data [30].

### Multivariate calibration methods

Multivariate techniques vary from each other in their manipulation of the raw data.

#### Partial least squares (PLS)

PLS is based on simultaneous decomposition of the concentration vector C and the predictor matrix X by using LVs latent variables [31]. To predict the optimum rank of number of PLS latent variables, cross validation (CV) is used [32]. The root mean square error of CV (RMSECV) is used to evaluate the performance of the model.

#### Supervised machine learning methods

Nowadays, regression machine learning methods are usually applied in analysis processes [33–35] as these methods can manipulate the data that contains noise and sources of non-linearity due to weighing errors as well as the concentrations not within the range of beer–lambert calibration [36, 37] and uncalibrated glassware.

##### Artificial neural networks (ANN)

It consists of artificial neurons linked by weights. The parameters of this network are reformed till its output agrees to the target. Several input/target pairs are utilized to confirm better training of the network and to provide trustworthy results [38].

A feed- forward back propagation (bpn) ANN and Encog ANN models were used in this work. Back-propagation ANN shows several advantages in signal processing and data reduction in addition to the prediction of spectra [39, 40]. Encog enables creation of a variety of ANN architectures by the language of Java programming [41].

Reduction of the inputs data is a key step to save the time for computing as well as to decrease the noise by choosing only data that are relevant to the components [42]. Data were compressed into scores using principal component analysis (PCA) and latent variables (PLS) that best described the data signal, then they were used as input data. Many transfer functions were tested including ones that can deal with expected non-linearity.

##### Support vector regression (SVR)

Its target is to obtain a multivariate regression function f (x) using the data in response matrix X (UV absorption) to calculate the target property (concentration). There are several parameters should be optimized to get successful SVR predictions such as cost (regularization constant) and epsilon (distance from actual values) or nu (lower bound of the number of SVs) in addition to gamma (kernel width parameter) in case of non-linear kernels. The kernel here was the RBF Gaussian Radial Basis Function to be fixable in dealing with expected non-linearities.

##### Extreme gradient boosting (XG Boost)

Using XGB model, several parameter values were tested. The parameter combinations which gave the best results were selected. XGB investigates several parameters eta, max_depth, and num_round. XGB uses CV cross-validation to choose the optimal XG boost parameter values and builds an XGB model using those values. Eta value(s) (0–1) used to control the learning rate of the gradient boosting. Max_depth value(s) used to specify the maximum depth allowed for the decision trees. Num_round value(s) used to specify how many rounds of tree creation to perform [43].

### Experimental design

#### Calibration and test sets solutions

**Fig. 2:**
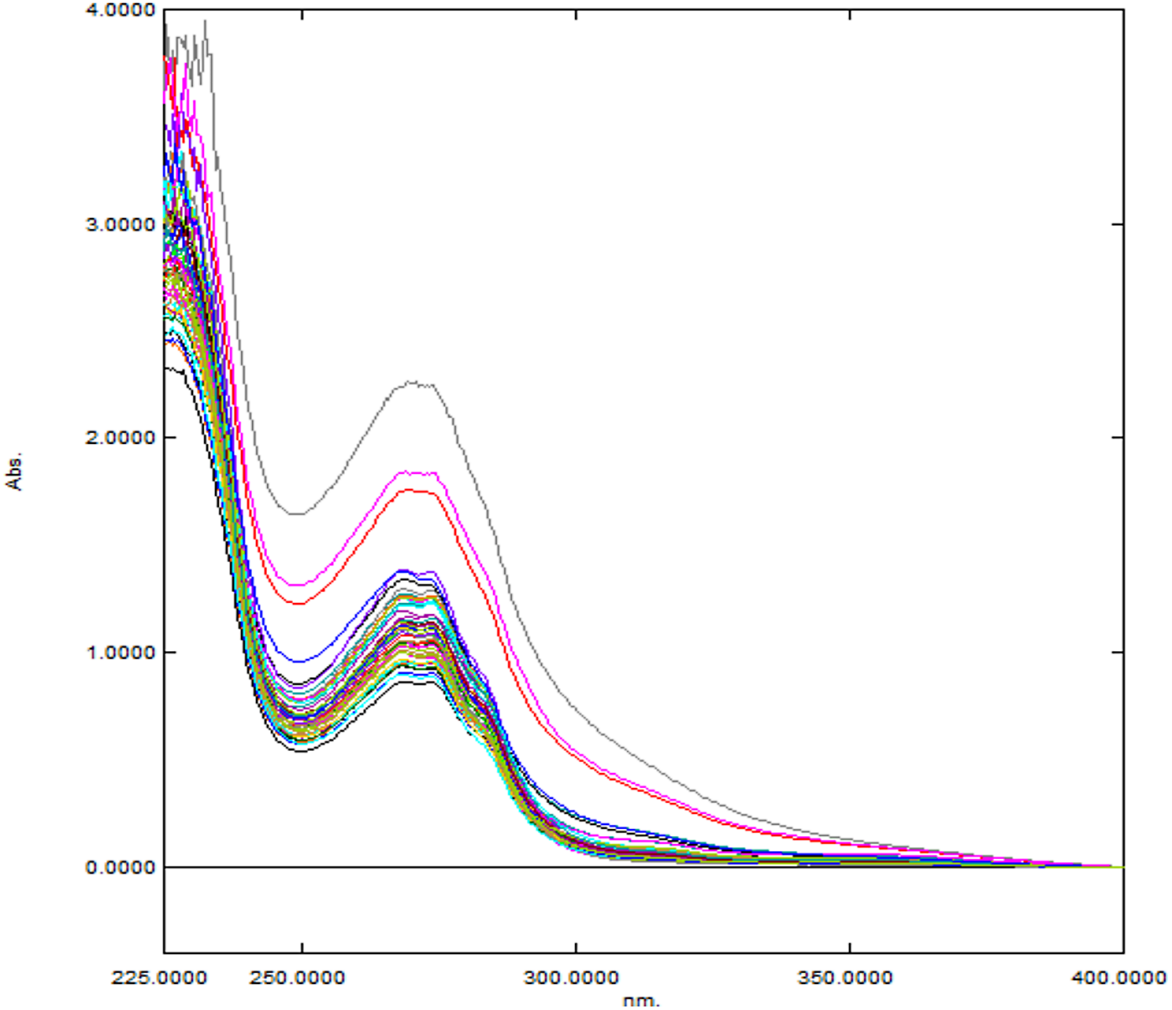
The overlay of UV spectra of training & test sets (the top highest three spectra) containing different concentration ratios of ALG, PIO and GLM in methanol

Multilevel multifactor design was implemented to build calibration set, a [44]. In this study, a calibration of 25 experiments in addition to 6 runs were measured to demonstrate instrumental stability [44]. Additional 12 experiments were measured to test the developed models [44]. The measured UV spectra were recorded over wavelength ranges (200–400) nm with 0.5 nm scanning resolution. Each sample was scanned three times, and the averaged spectra were incorporated in the developed models. The selected concentration ranges were (20–30) µg/mL for ALG, (24–36) µg/mL for PIO and (3.2–4.8) µg/mL for GLM. The different concentrations of training (M), instrumental stability (St) and test (T) sets solutions are presented in Table 1 and their UV spectra are illustrated in Fig. 2.

**Table 1:** The concentration design matrix for calibration (M), instrumental stability (St) and test (T) sets

No	Label	PIO	ALG	GLM	No	Label	PIO	ALG	GLM
1	St1	27.00	22.50	4.40	23	M19	24.00	25.00	4.40
2	M1	30.00	25.00	4.00	24	M20	30.00	27.50	4.40
3	M2	30.00	20.00	3.20	25	St5	27.00	22.50	4.40
4	M3	24.00	20.00	4.80	26	M21	33.00	27.50	3.60
5	M4	24.00	30.00	3.60	27	M22	33.00	22.50	3.20
6	M5	36.00	22.50	4.80	28	M23	27.00	20.00	3.60
7	St2	27.00	22.50	4.40	29	M24	24.00	22.50	4.00
8	M6	27.00	30.00	4.00	30	M25	27.00	25.00	3.20
9	M7	36.00	25.00	3.60	31	St6	27.00	22.50	4.40
10	M8	30.00	22.50	3.60	32	T1	25.00	26.50	3.67
11	M9	27.00	22.50	4.40	33	T2	31.00	26.10	3.27
12	M10	27.00	27.50	4.80	34	T3	24.00	20.60	3.65
13	St3	27.00	22.50	4.40	35	T4	30.00	27.86	3.47
14	M11	33.00	30.00	4.40	36	T5	32.50	21.98	3.65
15	M12	36.00	27.50	4.00	37	T6	27.00	26.10	3.27
16	M13	33.00	25.00	4.80	38	T7	25.50	23.35	3.06
17	M14	30.00	30.00	4.80	39	T8	27.00	29.23	3.06
18	M15	36.00	30.00	3.20	40	T9	32.50	24.73	3.86
19	St4	27.00	22.50	4.40	41	T10	25.50	27.86	4.67
20	M16	36.00	20.00	4.40	42	T11	27.00	21.98	3.27
21	M17	24.00	27.50	3.20	43	T12	33.50	26.10	4.65
22	M18	33.00	20.00	4.00					

**Fig. 3:**
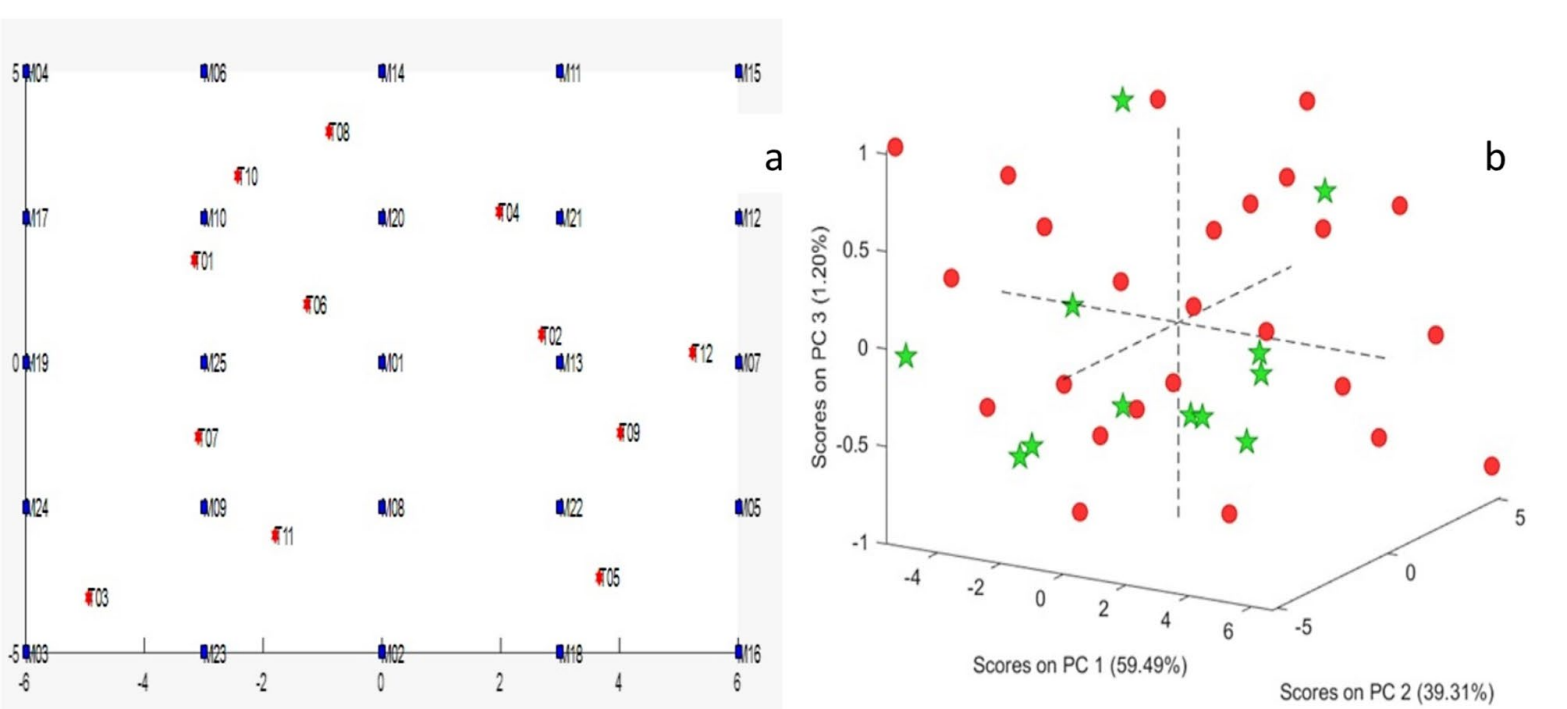
**a**) 2D scores plot for the mean centered 25 training samples (blue color) and 12 test samples (red color). (**b**) 3D scores plot for the mean centered 25 training samples (red circles) and 12 test samples (green stars)

The 2D plots for scores of the first two principal components (PCs) (Fig. 3a) and 3D plot for scores of the first three PCs (Fig. 3b) of the mean centered concentration matrix were obtained to ensure that the samples used in training set covered the mixture space properly as well as to ensure orthogonality and symmetry in addition to rotatability as illustrated in (Fig. 3a & b) [44]. The validity as well as the predictive ability of the developed models were tested by using a test set containing 12 samples (mixtures) that presented inside the concentration space of the design (Fig. 3a & b).

## Results and discussion

### Overview

This study investigated four multivariate calibration models PLS, ANN, SVR, and XG Boost to resolve the complex mixtures of PIO, ALG, and GLM, which presented highly convoluted and overlapping spectral data, particularly for ALG and PIO. The models aimed to address significant non-linearity issues within the spectra due to the components’ chemical properties and varying concentration ratios.

PLS struggled with non-linear data, especially for ALG and GLM, demonstrating the limitations of linear models in complex spectral environments. ANN required careful tuning to avoid overfitting, showing sensitivity to model complexity, while SVR, though robust against non-linear spectral noise, was still impacted by matrix effects in dosage forms, particularly with GLM. Preprocessing techniques like PCA and OSC played a crucial role in enhancing signal quality across all models, with non-linear methods benefiting most. The varying component ratios and significant spectral overlaps emphasized the need for effective feature selection to lessen interference.

Furthermore, the study provides guidance on model selection, highlighting the adaptability of XG Boost and SVR for routine pharmaceutical analysis, while recognizing the limitations of PLS and ANN, thus offering a comprehensive framework for future applications in complex spectral quantification. The XG Boost model consistently demonstrated superior performance, particularly in quantifying GLM, which was otherwise difficult for the other models due to extreme spectral overlap and matrix interference. Overall, XG Boost outperformed the other models in terms of accuracy, precision, and ability to manage non-linear spectral data.

### Spectral data variable selection and preprocessing

**Fig. 4:**
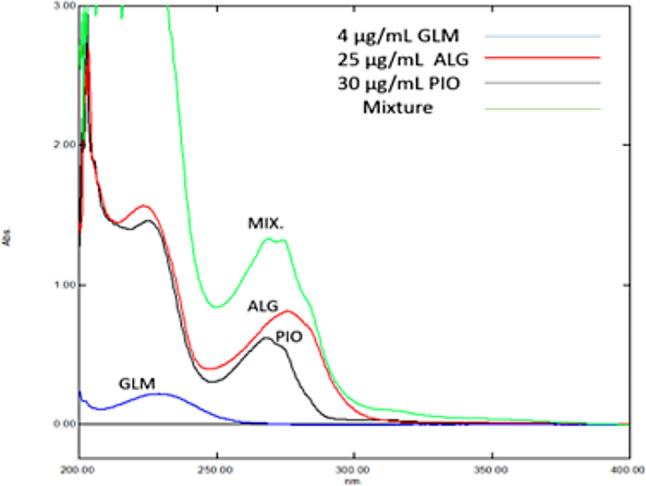
Overlay of zero order UV absorption spectra of ALG (red line), PIO (black line), GLM (blue line), and their mixture (green line) in methanol

The overlay of UV spectra of PIO, ALG and GLM showed the features of convoluted bands that are difficult to resolve that hinder the determination of each drug with univariate and most multivariate spectrophotometric methods. Severely and completely overlapped spectra along the informative wavelength range especially for ALG and PIO, similar chemical properties of considered drugs especially ALG and PIO and the wide range of components’ ratio in dosage forms led to complicated spectra with expected non-linear performance Fig. 4.

Additionally, it was noticed that the mixture components had a pivotal rule in the overall non-linearity of spectral characteristics for the analyzed drug mixtures due to design limitations. GLM made a modest contribution in the overall non-linearity (0, -1 and − 2) levels of the design used in calibration is lower than the proposed linearity range. ALG has greater influence due to only + 2 level of the design used in calibration being at the boundary of linearity range and all other design levels being beyond this range.

A blend of the problems defined above produced a distinctive mixture with a challenging non-linearity characteristic. While utmost of the published work is dealing with non-linearity problems were attentive mainly to mixtures with minor non-linearity problems (weighing faults, instrumental noise, and uncalibrated glassware), our work shows a resolution and quantification contest for four different chemometric models having different mathematical backgrounds to re solve mixtures that represents a thrilling non-linearity behavior.

The outside environment inevitably generates interfering noise, resulting in unreliable intensity of spectral signals. These problems combined make it tough for the quantitative analysis of complex mixed solutions and utterly restrict the application of spectral quantitative methodology.

To verify a quantitative analysis standard model, the work can be generally divided into three parts: spectral preprocessing followed by feature selection then multivariate calibration. Preprocessing methods that are frequently used to remove any source of variations such as base line shift, instrumental and environmental effects include mean centering (or auto scale), orthogonal scatter correction (OSC) and principal component analysis (PCA). For each model one or more of the proposed data processing techniques was/were adopted as described in the following sections.

Knowledge based variable selection or data reduction leaned on the selection of sensitive feature bands in each spectrum to extract the wavelength ranges that mostly affect the concentration of each drug. For each drug the following wavelength bands were selected; ALG and PIO (230.5–245 nm) and (260–300 nm), GLM (230.5–245 nm) and (300.5–380 nm) to help the developed models extract useful spectral information. Spectral resolution 0.5 nm was used to increase the depth of spectral information in the selected wavelength range. The dimensions of x-matrix for GLM were (25 × 190) and for PIO and ALG it was (25 × 111).

Subsequently, the multivariate calibration methods including linear principal component-based methods, e.g., partial least square (PLS) and the non-linear algorithms of machine learning regression methodologies, e.g., ANN (artificial neural networks) and SVR (support vector regression) as well as XG Boost (extreme gradient boosting) were developed and applied.

### Optimization of models parameters

#### PLS optimization

PLS was calculated with the SIMPLS algorithm [45] using mean centering preprocessing method for both ALG and PIO. The appropriate selection of the LVs number should be utilized in building the model is the secret to achieving accurate quantitation; the ideal number of LVs was 3 for ALG and PIO. The method evolved by Haaland and Thomas [31] was employed to ascertain the ideal number of factors, which entails choosing the model with the fewest components that produce a negligible difference between the minimum RMSECV and the equivalent RMSECV.

**Fig. 5:**
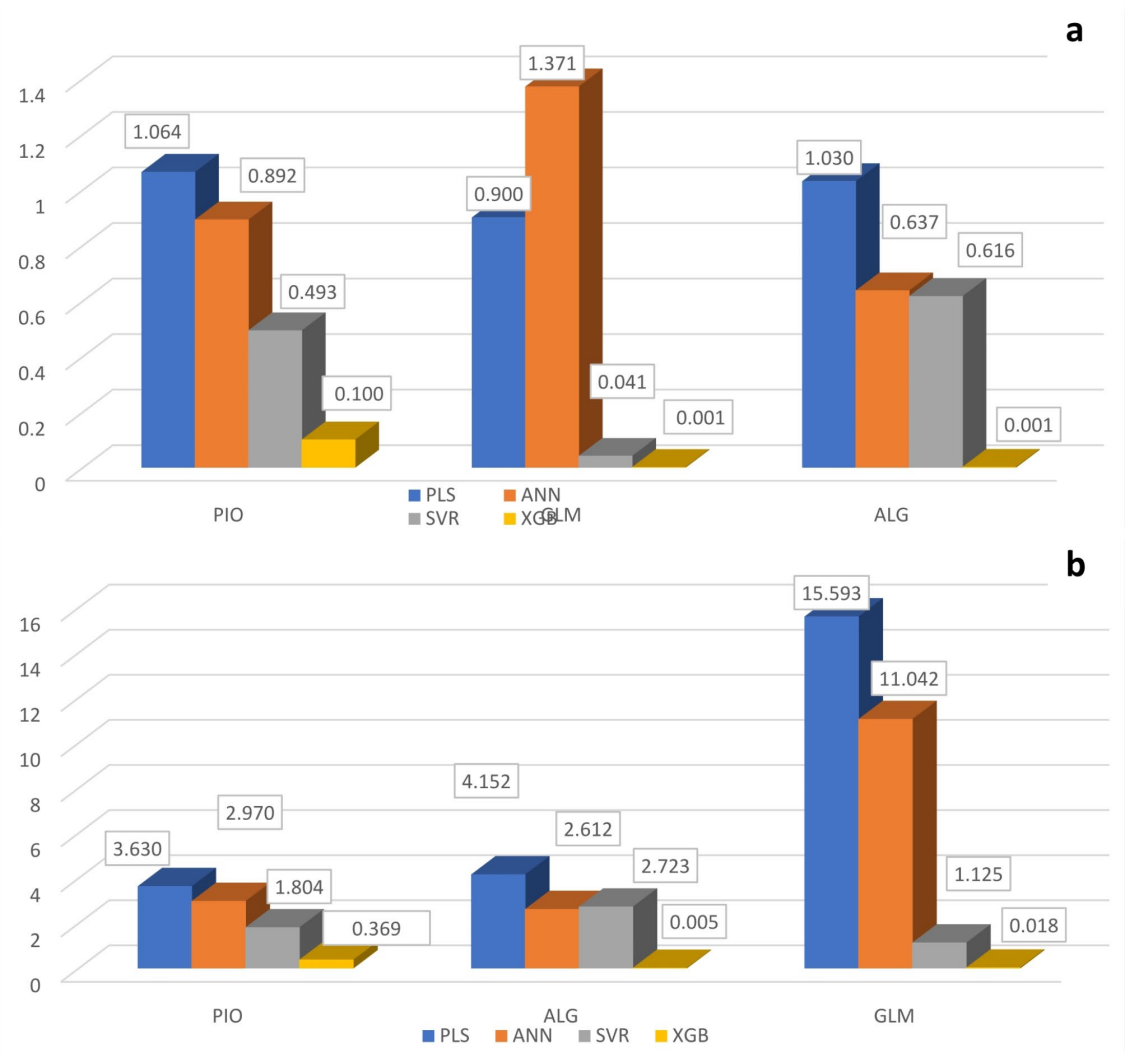
Bar plots for comparison of the RMSEP (**a**) and SD (**b**) values obtained by application of the four proposed models for the analysis of the test set

The expectation was not that PLS would outperform the other models. (SVR, ANN and XG boost) in the challenging determination of GLM and ALG. This could be explained by each compound’s unique spectrum properties. PLS performed the worst out of all the multivariate approaches that were examined because of how easily it could be calculated and how poorly it could handle nonlinearity in spectral data (Fig. 5).

#### ANN optimization

Low node counts for ANN were employed to prevent noisy data modeling, overfitting, and lengthy computer processing times [46]. Consequently, principal component analysis (PCA) was used to decrease the input matrix from 111 points to just four principal components of data. for ALG and using partial least square (PLS) from 111 points to only three latent variables (LVs) for PIO. Only the scores that accurately reflected the components under analysis were present in these components. For the components under investigation, a single hidden layer solved the problem; adding more hidden layers could lead to overfitting [39].

Optimization of the network architecture was achieved through trial-and-error, adjusting the number of hidden neurons based on RMSEP results (Table S2). Training was terminated when the RMSEP of the test set began to increase while that of the training set decreased, indicating potential overfitting. A learning rate of 0.125 was selected, balancing learning speed and model stability.

#### SVR optimization

SVR models were tailored for ALG, PIO, and GLM using nu-SVR with specific preprocessing and parameter adjustments to enhance predictive accuracy. Ortho Scatter Correction (OSC) was applied to reduce noise, and PCA was used for dimensionality reduction, maintaining key spectral features.

The radial basis function (RBF) kernel was selected for all compounds to capture non-linear relationships. Key parameters, including cost (C), nu (ν), and gamma, were optimized for each drug: ALG and PIO had C set to 100 and gamma at 0.1, while GLM used a lower C of 31.623 and a higher gamma of 3.162 to better resolve its complex spectra. The models demonstrated strong calibration with R² values of 0.972 for ALG, 0.988 for PIO, and 0.996 for GLM, confirming the efficacy of SVR in handling overlapping and non-linear spectral data. These adjustments enabled SVR to effectively predict complex pharmaceutical mixtures, particularly for challenging compounds like GLM (Table S3).

#### XG-boost optimization

XG-Boost models were tailored for GLM, ALG, and PIO using specific preprocessing methods. Mean Centering for GLM and Ortho Scatter Correction (OSC) for ALG and PIO as well as PLS compression to enhance signal quality.

The gradient boosting tree (gbtree) booster type was used, with learning rates (eta) set to 0.3 for GLM, 0.5 for ALG, and 0.1 for PIO to balance learning speed and precision. Maximum depths were adjusted to 4 for GLM, 6 for ALG, and 1 for PIO to capture the complexity of each dataset while avoiding overfitting. Each model achieved nearly perfect calibration with R² values of 0.9999 for both GLM and ALG, and 0.9996 for PIO. These optimized parameters enabled XG-Boost to deliver precise predictions, effectively handling non-linear and complex spectral data across all compounds (Table S4).

### Performance comparison and optimization of model parameters

For serving as a reference for pharmaceutical analysis and to assist in selecting an appropriate technique for the analysis of mixtures with difficult non-linear difficulties, four multivariate calibration models were employed to resolve and quantify the unique combination.

The proposed models could resolve PIO and ALG in the calibration set as well as test set were linear PLS, nonlinear ANN, nonlinear SVR and extreme gradient boosting (XG Boost) algorithm. For the determination of highly challenging GLM two models support vector regression (SVR) and extreme gradient boosting (XG Boost) algorithm were only able to resolve GLM in test set. Recovery percentage, RMSEP, and SD were used to evaluate the suggested models’ performance in terms of their capacity to predict and their intermediate precision (Table. S5, Fig. 5).

For four distinct chemometric models with disparate mathematical foundations, the work made a resolution and quantification challenge to cope with a mixture that represents an extreme in nonlinearity features. The outcome of this comparison can direct analysts toward appropriate models that can be applied in the future to determine each component in various dose forms with comparable issues.

The determination of GLM (with the lowest ratio) simultaneously with PIO and ALG is nearly impossible using univariate and many multivariate approaches as the ratios of these components are highly variable (30 PIO: 25 ALG: 4 GLM). GLM was determined in test set by two models SVR and XG-boost. While in dosage form GLM was determined by XG-boost model. SVR model didn’t give satisfactory assay results of GLM in dosage form due to matrix interference, but this was resolved by using XG-boost model which gave good, accepted assay results for GLM in dosage form.

ALG remarked with higher contribution in its combined dosage form spectra absorption data with the studied components, therefore nonlinearity performance was expected hence the ALG failed to be determined using linear PLS model in pharmaceutical dosage form.

In addition to overcoming issues with spectral overlaps and background noise, the XG Boost model can accurately determine data even in the absence of preprocessing techniques and extract richer spectrum information by combining significant characteristics.

To attain each model’s maximum performance and to prevent frequent multivariate issues including over fitting, poor fitting, and poor prediction performance, optimization of multivariate models is a crucial phase that benefits from analyst experiences. Every compound in this experiment was calibrated separately in every model. The fact that not all the examined compounds could be calibrated using all the evaluated models led to findings from the individual calibration being better than the global calibration. This was anticipated given the unusual variety in the component ratios of our research mixtures.

PLS was able to resolve PIO and ALG in both the calibration and test sets but struggled significantly with the highly challenging GLM due to its linear nature and inability to handle the complex non-linearity of the spectral data. PLS demonstrated moderate performance with PIO and ALG, showing reasonable R² values (e.g., R² = 0.85 for PIO and R² = 0.83 for ALG). However, it struggled significantly with GLM, reflected in its high RMSEP values (RMSEP = 1.37 for GLM) and Ratio of Performance to Deviation values (RPD = 3.41 for PIO, 4.03 for ALG, and 11.37 for GLM). These results indicate limited predictive accuracy and reliability, particularly in handling non-linear and overlapping spectral data.

ANN showed improved performance over PLS by leveraging non-linear regression capabilities, with improved R² values for PIO (R² = 0.90) and ALG (R² = 0.88) and lower RMSEP values (e.g., RMSEP = 0.90 for ALG). However, its RPD values (RPD = 3.33 for PIO, 4.10 for ALG, and 12.27 for GLM) were slightly better, suggesting some sensitivity to spectral noise and overfitting. While ANN provided better quantification of ALG and PIO compared to PLS, its performance remained suboptimal for GLM due to complexities in parameter optimization.

SVR demonstrated robustness in handling non-linearity and provided satisfactory results particularly with PIO (R² = 0.92, RMSEP = 0.49, RPD = 3.66) and ALG (R² = 0.91, RMSEP = 0.62, RPD = 4.42). Despite its strength in minimizing prediction errors, SVR encountered difficulties with GLM in dosage forms, despite its RPD of 27.44 for GLM. This indicates good predictive capability but also highlights challenges when dealing with matrix interferences in complex spectral data.

XG Boost consistently outperformed all other models across all compounds, achieving the highest R² values (e.g., R² = 0.98 for PIO, 0.99 for ALG, and 0.99 for GLM) and the lowest RMSEP (e.g., RMSEP = 0.10 for PIO, 0.001 for ALG, and 0.001 for GLM). Although, RPD values were not the highest among the models (RPD = 3.69 for PIO, 5.00 for ALG, and 18.00 for GLM), it successfully managed to quantify the analyzed compounds in their dosage forms. Its advanced non-linear regression approach allowed it to extract richer spectral information and achieve precise quantification even without extensive preprocessing or being affected by matrix interferences.

The comparison emphasizes that while PLS showed strong quantification performance for simpler cases, non-linear models like SVR and XG Boost offered superior handling of complex overlaps and noise, especially in mixtures with challenging non-linearity [28, 29].

The properties of each multivariate model employed in this study were variable. PLS is well recognized for its easier computation and conceptualization compared with other more advanced models such as XG-boost, SVR and ANN [47]. Because these models can do non-linear regression, they typically offer advantages over other multivariate models.

### Model generalization limitations

While acknowledging that while our study demonstrates the effectiveness of multivariate models such as XG Boost, SVR, and ANN in handling the complex spectral characteristics of the analyzed compounds, these findings may not fully extend to all pharmaceutical mixtures.

One limitation is the specific nature of the spectral data used in this study, which involved severe overlapping and non-linear spectral characteristics unique to the tested compounds (PIO, ALG, and GLM). The performance of these models might vary when applied to mixtures with different chemical properties, spectral profiles, or component ratios. For instance, models like SVR and XG Boost, which performed well in our study, may require re-optimization of parameters when dealing with other compounds or mixtures, particularly those with different matrix effects or spectral noise levels.

Additionally, while advanced models like XG Boost demonstrated robustness and accuracy, they are computationally intensive and sensitive to parameter tuning, which could affect their performance if not carefully managed in different contexts. The linear nature of PLS, although easier to implement, showed limitations in handling non-linearity, suggesting that it may not be the best choice for mixtures with high spectral complexity.

### Analysis of pharmaceutical products

**Fig. 6:**
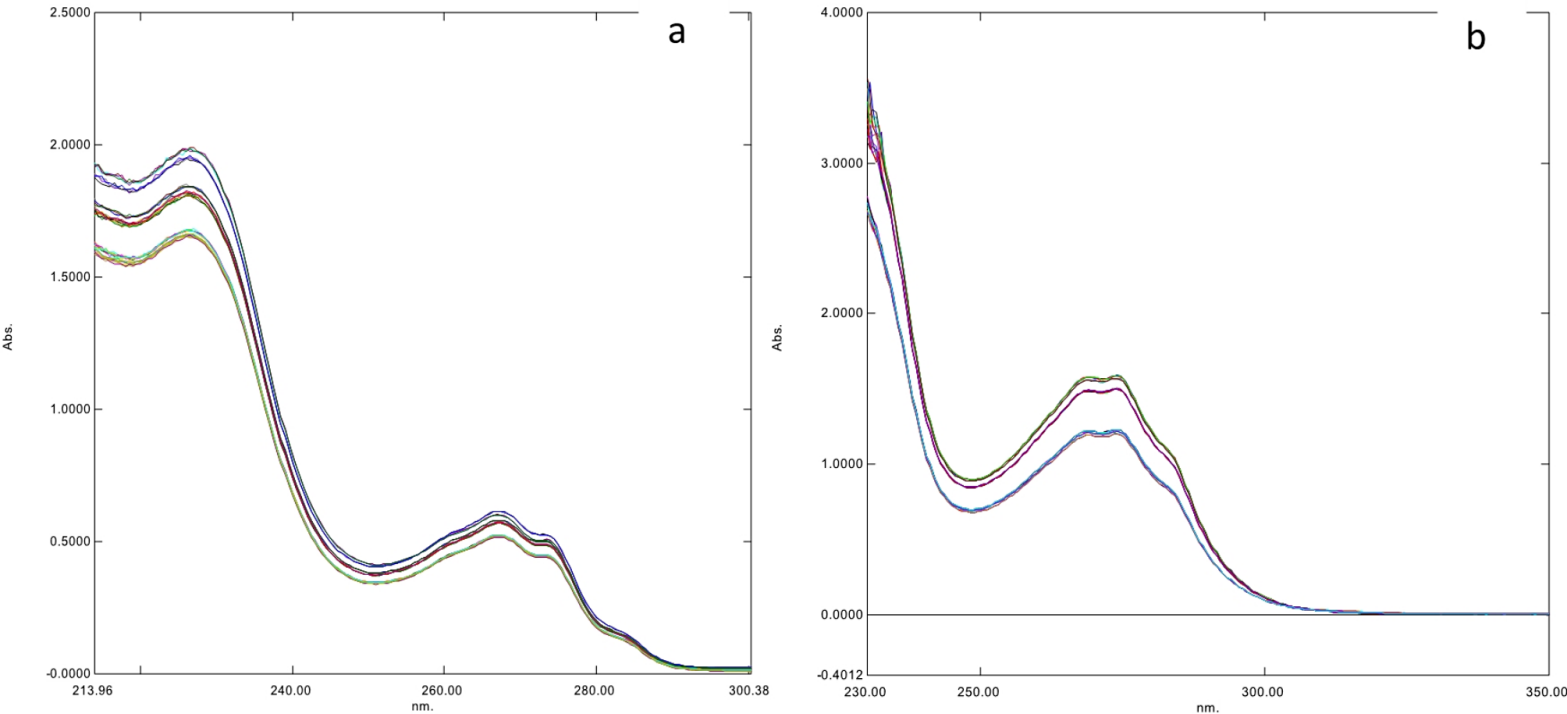
Overlay spectra of three different mixtures of (**a**) Piompride^®^ (4/30) tablet, (**b**) Prandglim plus^®^ tablets (25/30)

For the determination of (ALG with PIO) in Prandglim plus^®^ (25/30) and (PIO with GLM) in Piompride^®^ (4/30), different models with different parameters were developed and optimized. The overlay of UV absorption spectra of three different mixtures of Piompride^®^ (4/30) tablet are shown in Fig. 6 (a) and those of Prandglim plus^®^ tablets (25/30) are shown in Fig. 6 (b).

Three mixtures determined three times and the average were taken. The results as shown in Table 2. Good results were achieved for each drug in a satisfactory agreement with the labeled claims (Table 2).

**Table 2:** Results of assay of two dosage forms Prandglim plus^®^ and Piompride^®^ (4/30) using proposed models

Prandglim plus^®^ (25/30)											Piompride^®^ (4/30)												
**% Recovery**											**% Recovery**												
**Added conc.**		**PLS**		**ANN**		**SVR**		**XG-boost**				**Added conc.**			**PLS**			**ANN**		**SVR**		**XG-boost**	
**PIO**	**ALG**		**PIO**	**PIO**	**ALG**	**PIO**	**ALG**		**PIO**	**ALG**		**PIO**		**GLM**	**PIO**	**PIO**			**PIO**		**PIO**		**GLM**
27.00	22.50		100.72	102.20	95.15	100.15	104.84		99.96	103.91		30.00		4.00	102.25	100.29			101.30		101.92		100.33
33.00	27.50		104.48	105.70	99.04	100.35	105.17		102.09	104.99		33.00		4.40	100.87	99.72			99.57		100.30		98.68
36.00	30.00		106.15	100.46	97.46	98.09	104.55		100.00	99.35		36.00		4.60	95.29	93.29			92.89		93.24		97.36
Mean %		103.78		102.79	97.22	99.53	104.86	100.68		103.28					99.47		97.76			97.92	98.37		98.79
SD		2.78		2.67	1.96	1.25	0.31	1.22		3.00					3.68		3.88			4.44	4.62		1.49

SD; standard deviation

PIO was determined using the four proposed models in both test set and dosage forms spectra with superiority of (XG-Boost) algorithms results (SD and RMSEP) over other models.

ALG was also determined using the four proposed models in test set, but PLS model was failed to resolve and determine ALG in dosage form spectra as result of nonlinearity.

For GLM the only two models that could be used for determination were SVR and (XG-Boost) algorithms. In dosage form spectra only (XG-Boost) algorithms showed excellent results for determination of GLM, while SVR model was failed.

In summary, there are varying degrees of efficacy with which multivariate models may address nonlinearity issues in spectral data. The comparison based on the recovery percentage, standard deviations (S.D), and RMSEP of test set showed that the XG-boost model was the best model for the determination of the three components. Also, it was the only model used for the determination of GLM in dosage form spectra.

PLS performs the worst out of all the multivariate approaches that have been explored because of how easily it can be calculated and how poorly it can handle nonlinearity in spectral data when using ALG and GLM.

To test learning machine models’ well-known advantages over alternative models in the handling of spectral data with high nonlinearity, such as ANN, SVR, and XG-boost, were used. As seen by the lowest RMSEP and SD, the XG-boost model outperformed all other examined models for the determination of all suggested medications and outperformed other models in successfully extracting meaningful information from severe and complex nonlinear spectral data.

## Conclusion

Based on root mean squared error of prediction (RMSEP) and standard deviation (SD) data, four distinct multivariate calibration models PLS, SVR, ANN, and XG boost were compared in the current work to determine ALG, PIO, and GLM with varying degrees of accuracy and precision. The results showed that (XG Boost) algorithm had best performance regarding the determination of the three components in proposed mixtures with lowest (RMSEP) and standard deviation (SD) values. The SVR and XG Boost methods were the only two models able to determine the highly challenging GLM in test set. Non-linear models (ANN, SVR and XG boost) proven better performance, outstanding quantification power of spectral data with great complexity. The work introduced XG boost model as competent multivariate model that can be applied in situations where other multivariate models are not expected to perform well.

## Electronic supplementary material

Below is the link to the electronic supplementary material.

Supplementary Material 1

## Data Availability

Data is provided within the manuscript and supplementary information files. The datasets used and/or analyzed during the current study are available from the corresponding author on reasonable request.

## Abbreviations

IDF: International Diabetes Federation
ALG: Alogliptin benzoate
DPP-4: Dipeptidyl peptidase-4
PIO: Pioglitazone
PPAR: Peroxisome Proliferator-Activated Receptor
GLM: Glimepiride
PLS: Partial Least Square
ANN: Artificial Neural Networks
SVR: Support Vector Regression
XG Boost: Extreme Gradient Boosting
MC: Mean Centering
AS: Auto Scale
OSC: Orthogonal Scatter Correction
MSC: Multiplicative Scatter Correction
SNV: Standard Normal Varieties
RMSECV: Root Mean Square Error of Cross Validation
RMSEP: Root Mean Square Error of Prediction
RBF: Radial Basis Function
PCA: Principal Component Analysis
RMSEC: Root Mean Squared Error of Calibration
