# Supplementary material for: Decoding the synergy: unveiling gradient boosting regression model for multivariate quantitation of pioglitazone, alogliptin and glimepiride in pure and tablet dosage forms
Source: BMC Chem. 2024 Nov 29;18(1):237. doi: 10.1186/s13065-024-01351-8 (PMC11607924; doi:10.1186/s13065-024-01351-8)
Supplement: Supplementary file 1 — Supplementary Material 1 [file 13065_2024_1351_MOESM1_ESM.docx]

| **Equation no.** | **Equation form** | **Variables** | **Application model** |
| --- | --- | --- | --- |
| **1** | ***RMSECV =*** $\sqrt{PRESS/n}$ | ***n*** | PLS, SVR, XGB and ANN (BPN) |
| **2** | ***PRESS =* ∑ *(Y_pred_ – Y_true_ )^2^*** | ***Y_pred_, Y_true_*** | PLS, SVR, XGB and ANN (BPN) |
|  |  |  |  |
| **3** | $\boldsymbol{f}\left( \boldsymbol{x} \right)\boldsymbol{=}\sum_{\boldsymbol{i,j=1}}^{\boldsymbol{N}} \boldsymbol{(}\boldsymbol{\alpha}_{\boldsymbol{i}}\boldsymbol{-}\boldsymbol{\alpha}_{\boldsymbol{i}}^{\boldsymbol{*}}\boldsymbol{)}\left\langle\boldsymbol{\emptyset} \right.\boldsymbol{(}\boldsymbol{x}_{\boldsymbol{i}}\boldsymbol{).}\left. \boldsymbol{\emptyset}\left( \boldsymbol{x}_{\boldsymbol{j}} \right) \right\rangle\boldsymbol{+b}$ | ***X,* $\boldsymbol{\alpha}_{\boldsymbol{i}}$,** $\boldsymbol{\alpha}_{\boldsymbol{i}}^{\boldsymbol{*}}$ | SVR |
| **4** | *K ($\boldsymbol{x}_{\boldsymbol{i}}$,$\boldsymbol{x}_{\boldsymbol{i}}$)=exp*$\left( \frac{\boldsymbol{-}\left\Vert\boldsymbol{x}_{\boldsymbol{i}}\boldsymbol{-}\boldsymbol{x}_{\boldsymbol{j}} \right\Vert^{\boldsymbol{2}}}{\boldsymbol{2}\boldsymbol{\sigma}^{\boldsymbol{2}}} \right)$ | *K,* σ | SVR |
| **5** | ***RMSEP =*** $\sqrt{\frac{\sum_{i=1}^{n} ( \mathop{\mathop{c_{i}}\limits^{\wedge} - c_{i}- bias)^{2}}\limits}{n-1}}$ | ***c_i_*, ĉ*_i_*, *n*** | PLS, SVR, ANN and XGB |
| **6** | ***bias* =** $\frac{\sum_{i=1}^{n} \mathop{\mathop{c_{i}}\limits^{\wedge} - c_{i}}\limits}{n}$ | ***c_i_*, ĉ*_i_*, *n*** | PLS, SVR, ANN and XGB |

**Table .S1:** List of different equations used for selection of optimum parameter conditions and to compare the different models

***n***, is the number of training samples; ***Y_pred_*** and ***Y_true_*** are predicted and true concentrations in μgml^-1^, respectively; ***X*** *(i,j)* is a matrix of the UV absorbance spectra for the ***j*** variables (wavelengths in our case); ***i*** is the samples; ***C*** *(i, n)* is a matrix of the concentration profiles for the *n* components; ***K*** is a matrix of the pure component signals (spectra at unit concentrations in our case); $\boldsymbol{\alpha}_{\boldsymbol{i}}$ and $\boldsymbol{\alpha}_{\boldsymbol{i}}^{\boldsymbol{*}}$ are the Lagrange multipliers that satisfy the constraint 0≤$\alpha_{i}$ and $\alpha_{i}^{*}$≤*C*; σ is the kernel width parameter; *c_i_* is the reference concentration; ĉ*_i_* is the calculated concentration.

**Table. S2**: Optimized parameters of different ANN models for ALG and PIO

| **ANN** | | |
| --- | --- | --- |
| Drug | **ALG** | **PIO** |
| Algorithm type | ENCOG | BPN algorithm |
| Preprocessing | OSC, 4 components | Mean Center |
| Architecture | 4-19-1 | 3-4-1 |
| Hidden neurons number | 19 | 4 |
| X-block Compression: | PCA with 4 components | PLS with 3 components |
| Learn rate | 0.125 | 0.125 |
| Learn cycles | 20 | 20 |
| Transfer functions | sigmoid | sigmoid |
| RMSEC | 0.616 | 1.254 |
| RMSECV | N.A. | 1.638 |
| R^2 Cal | 0.964 | 0.902 |

RMSEC; Root mean squared error of calibration, RMSECV; Root mean squared error of cross validation, R^2 Cal; coefficients of determination of calibration, PCA; principal components analysis, N.A.; not applicable.

**Table. S3:** Optimized parameters of different SVR models for ALG, PIO and GLM

| **nu-SVR** | | | |
| --- | --- | --- | --- |
| Drug | ALG | PIO | GLM |
| Preprocessing: | OSC | OSC | OSC |
|  | components = 4, #iters = 0 | components = 4, #iters = 0 | components = 4, #iters = 1 |
| X-block Compression: | PCA with 5 components | PCA with 4 components | PCA with 4 components |
| SVM kernel type: | radial basis function | radial basis function | radial basis function |
| Cost | 100 | 100 | 31.623 |
| nu | 0.5 | 0.2 | 0.2 |
| Gamma | 0.1 | 0.1 | 3.162 |
| Number of SVs | 23 | 10 | 13 |
| RMSEC | 0.553 | 0.443 | 0.036 |
| RMSECV | 2.177 | 3.162 | 0.782 |
| R^2 Cal: | 0.972 | 0.988 | 0.996 |
|  |  |  |  |

RMSEC; Root mean squared error of calibration, RMSECV; Root mean squared error of cross validation, nu; lower bound of the number of SVs, cost; regularization constant and gamma; kernel width parameter, R^2 Cal; coefficients of determination of calibration. OSC; ortho scatter correction. PCA; principal component analysis.

**Table.S4:** Optimized parameters of different XG boost models for three compounds

| PIO | ALG | GLM | PIO | | ALG | | GLM | | PIO | | ALG | | | GLM | | PIO | | ALG | | PIO | | ALG | |
| --- | --- | --- | --- | --- | --- | --- | --- | --- | --- | --- | --- | --- | --- | --- | --- | --- | --- | --- | --- | --- | --- | --- | --- |
|  |  |  | F | %R | F | %R | F | %R | F | %R | F | %R | | F | %R | F | %R | F | %R | F | %R | F | %R |
| 25 | 26.5 | 3.7 | 25.20 | 100.82 | 26.54 | 100.15 | 3.70 | 100.00 | 25.66 | 102.62 | 26.00 | | 98.10 | 3.74 | 98.91 | 25.40 | 101.59 | 26.54 | 100.15 | 25.45 | 101.81 | 25.85 | 97.55 |
| 31 | 26.0 | 3.3 | 30.82 | 99.43 | 26.00 | 100.01 | 3.30 | 100.00 | 30.33 | 97.85 | 25.96 | | 99.84 | 3.31 | 99.61 | 30.21 | 97.46 | 26.00 | 100.01 | 29.58 | 95.42 | 27.25 | 104.81 |
| 24 | 20.5 | 3.7 | 24.03 | 100.11 | 21.45 | 104.65 | 3.70 | 100.01 | 24.35 | 101.44 | 21.91 | | 106.87 | 3.65 | 101.45 | 24.08 | 100.34 | 21.45 | 104.65 | 23.69 | 98.71 | 20.92 | 102.05 |
| 30 | 28.0 | 3.5 | 29.95 | 99.84 | 28.63 | 102.25 | 3.50 | 100.00 | 30.00 | 99.99 | 28.30 | | 101.08 | 3.54 | 98.73 | 29.41 | 98.04 | 28.63 | 102.25 | 29.09 | 96.98 | 26.93 | 96.17 |
| 32.5 | 21.98 | 3.7 | 32.57 | 100.22 | 21.23 | 96.60 | 3.70 | 100.01 | 33.23 | 102.25 | 21.27 | | 96.77 | 3.70 | 99.88 | 34.24 | 105.37 | 21.23 | 96.60 | 34.23 | 105.32 | 21.64 | 98.47 |
| 27 | 26.0 | 3.3 | 27.03 | 100.11 | 25.24 | 97.06 | 3.30 | 100.03 | 27.51 | 101.88 | 25.07 | | 96.41 | 3.27 | 100.99 | 27.97 | 103.60 | 25.24 | 97.06 | 27.98 | 103.64 | 25.69 | 98.82 |
| 25.5 | 23.5 | 3.1 | 25.39 | 99.58 | 23.66 | 100.68 | 3.10 | 99.97 | 25.50 | 99.99 | 23.72 | | 100.92 | 3.10 | 99.99 | 26.98 | 105.82 | 23.66 | 100.68 | 27.09 | 106.25 | 25.80 | 109.78 |
| 27 | 29.0 | 3.1 | 26.99 | 99.96 | 28.81 | 99.36 | 3.10 | 99.97 | 26.66 | 98.74 | 28.89 | | 99.62 | 3.07 | 100.95 | 26.65 | 98.71 | 28.81 | 99.36 | 26.69 | 98.85 | 28.86 | 99.53 |
| 32.5 | 24.5 | 3.9 | 32.50 | 100.01 | 25.60 | 104.50 | 3.90 | 100.00 | 31.84 | 97.98 | 24.87 | | 101.50 | 3.97 | 98.29 | 31.80 | 97.85 | 25.60 | 104.50 | 31.36 | 96.51 | 24.26 | 99.02 |
| 25.5 | 28.0 | 4.7 | 25.43 | 99.73 | 27.01 | 96.48 | 4.70 | 100.01 | 25.89 | 101.53 | 27.63 | | 98.69 | 4.65 | 100.97 | 25.36 | 99.45 | 27.01 | 96.48 | 25.53 | 100.12 | 26.75 | 95.53 |
| 27 | 21.98 | 3.3 | 26.93 | 99.73 | 22.25 | 101.25 | 3.30 | 99.98 | 26.34 | 97.57 | 22.60 | | 102.85 | 3.36 | 98.22 | 27.05 | 100.20 | 22.25 | 101.25 | 27.37 | 101.36 | 22.85 | 103.96 |
| 33.5 | 26.0 | 4.7 | 33.37 | 99.61 | 26.30 | 101.16 | 4.70 | 100.01 | 33.51 | 100.03 | 26.37 | | 101.41 | 4.73 | 99.26 | 34.76 | 103.76 | 26.30 | 101.16 | 35.06 | 104.64 | 24.76 | 95.24 |
|  |  |  | Mean | 99.93 |  | 100.34 |  | 100.00 |  | 100.16 |  | | 100.34 |  | 99.77 |  | 101.02 |  | 100.34 |  | 100.80 |  | 100.08 |
|  |  |  | SD | 0.37 |  | 2.61 |  | 0.02 |  | 1.80 |  | | 2.72 |  | 1.13 |  | 2.97 |  | 2.61 |  | 3.63 |  | 4.15 |
|  |  |  | RMSEP | 0.10 |  | 0.64 |  | 0.00 |  | 0.49 |  | | 0.62 |  | 0.04 |  | 0.89 |  | 0.64 |  | 1.06 |  | 1.03 |

**Table.S5:** Results of independent test set using different models

|  | GLM | ALG | PIO | |
| --- | --- | --- | --- | --- |
| Preprocessing | Mean Center | OSC | OSC | |
| X-block Compression | PLS with 5 component(s) | PLS with 4 component(s) | | PLS with 4 component(s) |
| Type | booster = gbtree | booster = gbtree | gbtree | |
| eta | 0.3 | 0.5 | 0.1 | |
| max_depth | 4 | 6 | 1 | |
| num_round | 500 | 500 | 500 | |
| Cross validation | venetian blinds w/ 10 splits and blind thickness = 1 | custom (user) split | custom (user) split | |
| RMSEC | 0.0009 | 0.0007 | 0.0778 | |
| RMSECV | 0.4351 | 0.9241 | 0.3648 | |
| R^2 Cal | 0.9999 | 0.9999 | 0.9996 | |

RMSEC; Root mean squared error of calibration, RMSECV; Root mean squared error of cross validation, R^2Cal; coefficients of determination of calibration, R^2 CV; coefficients of determination of cross validation. PLS; partial least square.
